# Effects of neurostimulation for advanced Parkinson’s disease patients on motor symptoms: A multiple-treatments meta-analysas of randomized controlled trials

**DOI:** 10.1038/srep25285

**Published:** 2016-05-04

**Authors:** Cheng-Long Xie, Bei Shao, Jie Chen, Yi Zhou, Shi-Yi Lin, Wen-Wen Wang

**Affiliations:** 1 The center of Traditional Chinese Medicine, The Second Affiliated Hospital & Yuying Children’s Hospital of Wenzhou Medical University, Wenzhou 325027, China; 2 Department of Neurology, The First Affiliated Hospital of Wenzhou Medical University, Wenzhou 325000, China

## Abstract

Deep brain stimulation (DBS) is the surgical procedure of choice for patients with advanced Parkinson disease (PD). We aim to evaluate the efficacy of GPi (globus pallidus internus), STN (subthalamic nucleus)-DBS and medical therapy for PD. We conducted a systematic review and multiple-treatments meta-analysis to investigate the efficacy of neurostimulation and medical therapy for PD patients. Sixteen eligible studies were included in this analysis. We pooled the whole data and found obvious difference between GPi-DBS versus medical therapy and STN-DBS versus medical therapy in terms of UPDRS scores (Unified Parkinson’s Disease Rating Scale). Meanwhile, we found GPi-DBS had the similar efficacy on the UPDRS scores when compared with STN-DBS. What is more, quality of life, measured by PDQ-39 (Parkinson’s disease Questionnaire) showed greater improvement after GPi-DBS than STN-DBS. Five studies showed STN-DBS was more effective for reduction in medication than GPi-DBS. Overall, either GPi-DBS or STN-DBS was an effective technique to control PD patients’ symptoms and improved their functionality and quality of life. Meanwhile, the UPDRS scores measuring parkinsonian symptoms revealed no significant difference between GPi-DBS and STN-DBS. STN-DBS was more effective for reduction in medication than GPi-DBS. Alternatively, GPi-DBS was more effective for improving the PDQ-39 score than STN-DBS.

Parkinson’s disease (PD) is a progressive debilitating neurodegenerative disease that affects dopaminergic neurotransmission, resulting in bradykinesia, rest tremor, and rigidity *et al.*^1^. After an initial honeymoon period, during which there is a sustained response to dopaminergic treatment, beneficial effects are hampered by levodopa (L-dopa)-induced motor complications. Advanced PD patients often show rapid, seemingly unpredictable swings between mobility (the on phase), usually with L-dopa-induced dyskinesias, and immobility (the off phase)^2^. Many of these patients suffer from unsatisfactorily to adjustment in pharmacological treatments, progressively compromising quality of life^3^. Deep brain stimulation (DBS) is an accepted therapy which is widely used to treat advanced PD patients and, increasingly, other movement, epilepsy and psychiatric disorders by targeting different brain structures, but its accurate mechanism of action has been elusive^4^. Although approved as a treatment for PD for over a decade, the underlying mechanism of DBS remains unclear. Different mechanisms have been proposed to explain the therapeutic actions of DBS^5^.

Well-established in the literatures and in practices, DBS of either the subthalamic ucleus (STN) or globus pallidus interna (GPi) is an increasingly common therapy for mid- to late-stage PD^6^. With optimized stimulation settings, DBS typically lessens the motor symptoms of tremor, limb rigidity, bradykinesia, and akinesia^7^. It is often used after the disease has been present for exceed 10 years, when quality of life, psychosocial competence, and professional activity are already severely impaired^8^. Recent publications have suggested that DBS is superior to best medical therapy to alleviate motor symptoms and improve quality of life or even discontinuation of medications^9^. Moreover, STN-DBS and GPi-DBS maybe are similarly effective in improving motor function and quality of life for patients with PD^6^. However, there still has been an ongoing debate GPi or STN who is the more preferable target for treatment of PD symptoms^10^. Numerous studies have documented significant improvement in motor symptoms and quality of life after STN-DBS^11^. On the other hand, several studies also have indicated similar effects of GPi-DBS on motor and non-motor symptoms. Reducing dyskinesia is a major goal of PD treatment by GPi-DBS, which ameliorates the off-period dystonia, cramps, and sensory symptoms associated with advanced PD^12^.

In the past decade, several systematic reviews and meta-analyses assessing the effects of GPi-DBS, STN-DBS and medical therapy on patients suffering from PD have been published. Galit *et al.* reported that STN-DBS improved motor activity and activities of daily living in advanced PD, which was result from non-randomised, prospective studies^13^. George *et al.* pointed out that DBS could ameliorate postural instability and gait disability. Further, the results suggested that GPi may be a superior site to STN for sustaining gait and posture function in combination with L-dopa^7^. Recently, Perestelo-Pérez L *et al.* showed that DBS plus medication versus medication alone significantly improved patients’ symptoms, functionality and quality of life based on six studies^14^. In this study, we reported a comprehensive overview of all randomized controlled trials (RCTs) that compared GPi-DBS, STN-DBS and medical therapy in terms of efficacy and acceptability in the advanced PD patients. Therefore, standard direct multiple-treatments meta-analyses were used to compared GPi-DBS versus STN-DBS versus medical therapy in advanced PD patients. We aimed to provide a clinically useful summary of the results of the multiple-treatments meta-analyses that could be used to guide treatment decisions.

## Results

### Study inclusion and basic characteristics of studies

**Figure 1:**
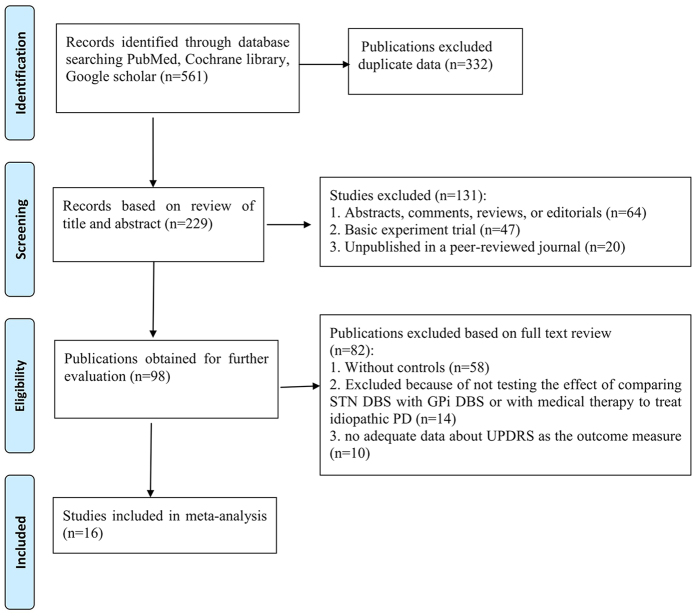
PRISMA 2009 Flow Diagram.

We identified 561 references, from which we excluded 332 due to the duplicates. After screening the titles and abstracts, 131 were excluded because they failed to meet the inclusion criteria. By reading the full text of the remaining 98 articles, 58 studies were excluded because a result of without control groups, 14 were excluded because of not testing the effects of comparing STN-DBS with GPi-DBS or with medical therapy to treat idiopathic PD, 10 studies were eliminated due to no adequate data about UPDRS as the outcome measure. Ultimately, just leaving 16 qualified studies satisfied the pre-established inclusion criteria^10^,^15^,^16^,^17^,^18^,^19^,^20^,^21^,^22^,^23^,^24^,^25^,^26^,^27^,^28^,^29^ (Fig. 1).

Sixteen RCTs, with a total of 2186 participants, met the inclusion criteria and were included in this review. Studies were conducted in America (8/16, 50.0%), Italy (2/16, 12.5%), Germany (3/16, 18.6%), Switzerland (1/16, 6.3%), UK (1/16, 6.3%) and Netherlands (1/16, 6.3%), respectively. The mean age of those included studies was relatively elder at about 55 years, most were in Hoehn & Yahr stage II or more and there were slightly more males than females. The number of participants randomized into the 16 trials included in this meta-analysis ranged from 20 to 366 participants. Among all studies, GPi-DBS versus medical therapy in 3 studies, STN-DBS versus medical therapy in 5 studies, and remaining 8 studies examined GPi-DBS versus STN-DBS. Meanwhile, the time of follow up was ranged from 6 months to 3 years. In terms of outcome measures, UPDRS part I was observed in 6 studies; UPDRS part II (on phase or off phase) was observed in 10 or 6 studies, respectively; UPDRS part III (on phase or off phase) was reported in 16 and 9 studies, respectively; UPDRS part IV was showed in 7 studies. Moreover, PDQ-39 and Levodopa equivalent dose (LED, pre- and post-surgery) were reported as outcome measures in 9 and 11 studies, respectively. The basic characteristics of the 16 selected studies were summarized in Table 1.

### Risk of bias

Table 2 showed the risk of bias of the included trials. All trials described the method of randomization used (e.g. random number table, computer generated). Fourteen trials gave information that allowed the assessment of whether an adequate concealment of allocation procedure was used. Twelve studies reported the blinding of participants. All trials described intention-to-treat analyses (ITT) and reported follow up data. Therefore, all of the included trials were deemed to have a low risk of bias.

### UPDRS: GPi-DBS versus medical therapy and STN-DBS versus medical therapy

**Figure 2:**
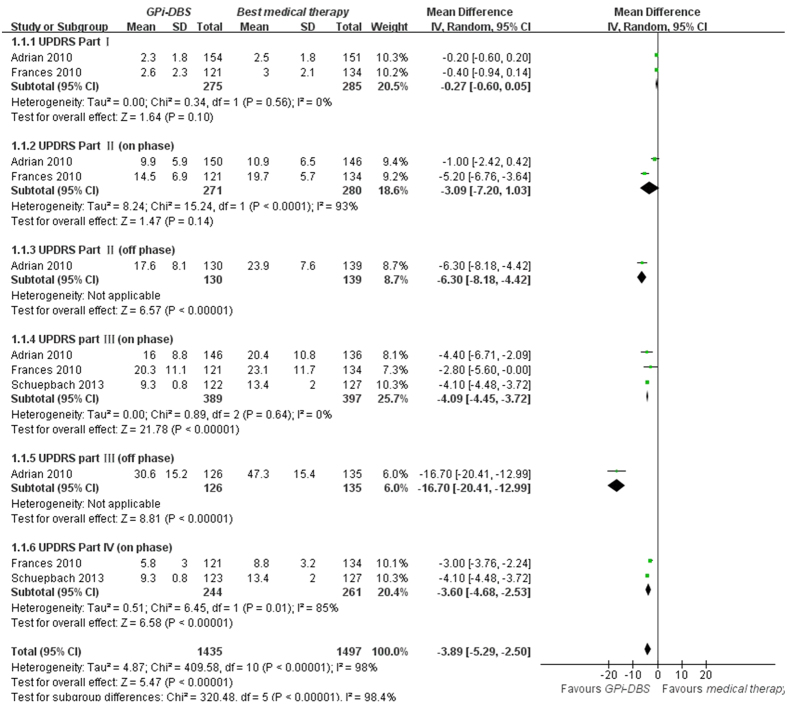
Effect sizes of GPi-DBS vs medical therapy for UPDRS Part I, UPDRS Part II (on phase), UPDRS Part II (off phase), UPDRS part III (on phase), UPDRS part III (off phase) and UPDRS Part IV (on phase). UPDRS: Unified Parkinson’s disease Rating Scale.

**Figure 3:**
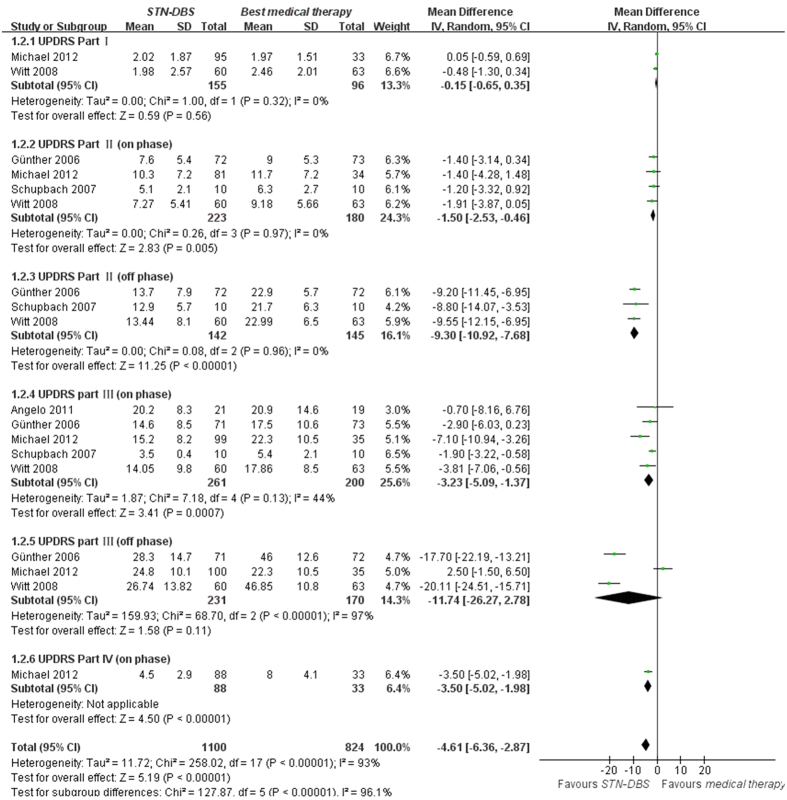
Effect sizes of STN-DBS vs medical therapy for UPDRS Part I, UPDRS Part II (on phase), UPDRS Part II (off phase), UPDRS part III (on phase), UPDRS part III (off phase) and UPDRS Part IV (on phase).

UPDRS Part I data were available from 2 trials of GPi-DBS compared with medical therapy. We pooled the data and found no significant difference between two groups (p = 0.10, WMD (weighted mean difference) = −0.27, 95% confidence interval (CI): −0.60 to 0.05, Fig. 2). In terms of UPDRS Part II in the on-medication phase, no statistical difference was obtained between GPi-DBS and medical therapy (p = 0.14, WMD = −3.09, 95% CI: −7.20 to 1.03, Fig. 2). However, in the off-medication phase, the difference showed significant discrepancy in favor of GPi-DBS (p < 0.00001, WMD = −6.30, 95% CI: −8.18 to −4.42, Fig. 2). Data on the clinical associated UPDRS part III score in the on-medication phase showed the overall effect of GPi-DBS was more effective than medical treatment (p < 0.00001, WMD = −4.09, 95% CI: −4.45 to −3.72, Fig. 2). Meanwhile, a statistical significant effect in favor of the GPi-DBS was obtained in the off-medication phase (p < 0.00001, WMD = −16.70, 95% CI: −20.41 to −12.99, Fig. 2). Finally, two studies provided data on the UPDRS Part IV showed obvious effect in favor of the GPi-DBS compared with medical therapy (p < 0.00001, WMD = −3.60, 95% CI: −4.68 to −2.53, Fig. 2). Moreover, we pooled the whole data to process and found significant difference when STN-DBS compared with medical therapy according to UPDRS Part II (on phase, p = 0.005, WMD = −1.50, 95% CI: −2.53 to −0.46, Fig. 3), UPDRS Part II (off phase, p < 0.00001, WMD = −9.30, 95% CI: −10.92 to −7.68, Fig. 3), UPDRS part III (on phase, p = 0.0007, WMD = −3.23, 95% CI: −5.09 to −1.37, Fig. 3) and UPDRS Part IV (on phase, p < 0.00001, WMD = −3.50, 95% CI: −5.02 to −1.98, Fig. 3), respectively. On the contrary, there was no statistical difference between STN-DBS and medical therapy in terms of UPDRS Part I (p = 0.56, Fig. 3) and UPDRS part III (off phase, p = 0.11, Fig. 3). However, we should interpret the pool results prudently due to the limited numbers of studies.

### UPDRS: GPi-DBS versus STN-DBS

**Figure 4:**
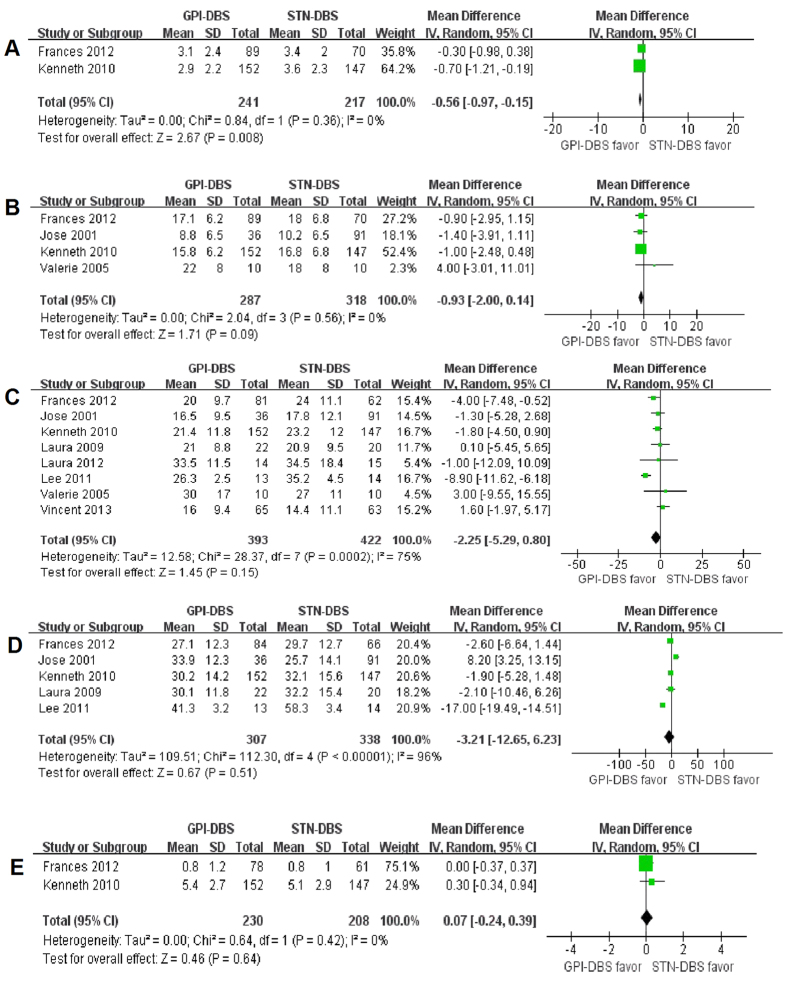
Effect sizes of GPi-DBS vs STN-DBS for (**A**) UPDRS Part I (on phase); (**B**) UPDRS Part II (on phase); (**C**) UPDRS part III (on phase); (**D**) UPDRS part III (off phase) and (**E**) UPDRS Part IV (on phase).

There were 8 studies examined GPi-DBS versus STN-DBS comprising a total sample size of 838 PD patients (403 GPi-DBS, 435 STN-DBS). Among them, two studies compared GPi-DBS with STN-DBS were included in the efficacy analysis, the effect size of UPDRS Part I score is higher in GPi-DBS than STN-DBS (p = 0.008, WMD = −0.56, 95% CI: −0.97 to −0.15, Fig. 4A). However, use of GPi-DBS did not yield any significant improvement over STN-DBS in the UPDRS Part II score (p = 0.09, WMD = −0.93, 95% CI: −2.00 to 0.14, Fig. 4B). Moreover, no significant differences were observed between GPi-DBS and STN-DBS in terms of UPDRS part III in the on-medication or off-medication phase, respectively (p = 0.15, p = 0.51, Fig. 4C,D, respectively). Finally, two studies provided data on the UPDRS Part IV still showed no statistical difference in favor of the GPi-DBS compared with STN-DBS (p = 0.64, WMD = 0.07, 95% CI: −0.24 to 0.39, Fig. 4E). Overall, GPi-DBS or STN-DBS equally improved PD symptoms, measured by the UPDRS.

### PDQ-39 and LED

**Figure 5:**
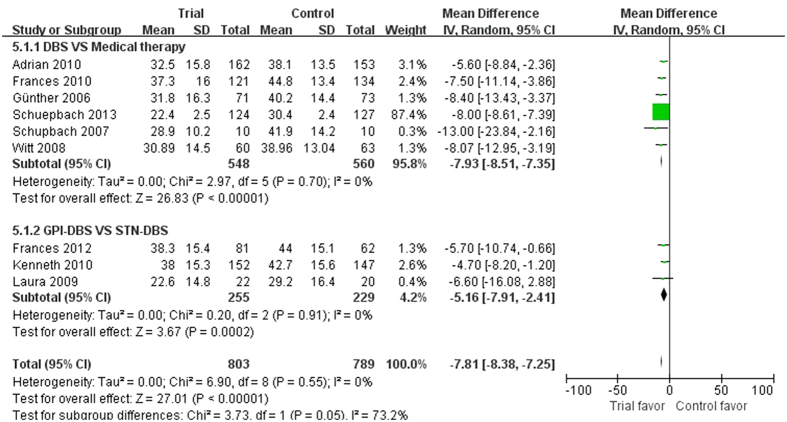
Effect sizes of DBS plus medical therapy vs medical therapy for PDQ-39 (Parkinson’s disease Questionnaire).

**Figure 6:**
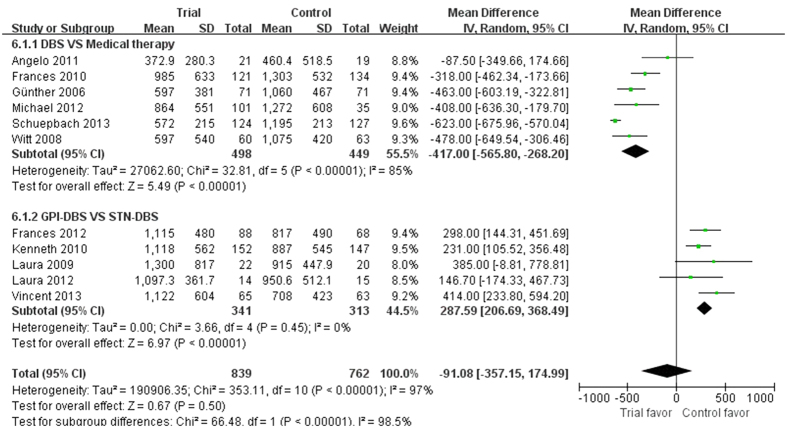
Effect sizes of DBS plus medical therapy vs medical therapy for LED (Levodopa equivalent dose).

Nine studies reported the PDQ-39 as the outcome measure with 1592 participants included in the analysis, from which six studies compared DBS with medical therapy found a significant difference in favor of DBS (p < 0.00001, WMD = −7.93, 95% CI: −8.51 to −7.35, Fig. 5). In addition, remaining three studies showed significant statistical difference between GPi-DBS and STN-DBS (p = 0.0002, WMD = −5.16, 95% CI: −7.91 to −2.41, Fig. 5). Among them, Laura *et al.*^21^ showed all patients endorsed better overall quality of life (QoL) after surgery. However, GPi patients improved more than STN patients (38% vs 14%, respectively; p = 0.03). Patients reported better PDQ-39 on subscales of mobility, activities of daily living (ADLs), emotional well-being, stigma, cognition and discomfort, but not on those of social support and communication. However, Kenneth *et al.*^23^ demonstrated that the QoL improved on six of eight subscales of the PDQ-39 in the GPi and STN-DBS groups after 24 months. Nevertheless, none of the between-group differences were significant (p > 0.05). Frances *et al.*^27^ only reported all PDQ-39 subscales improved following GPi and STN-DBS. Data on the L-dopa or levodopa-equivalent doses were extracted from eleven studies. Six studies showed considerable decrease in the medication dose of the DBS group compared to medical therapy group (p < 0.00001, WMD = −417.00, 95% CI: −565.80 to −268.20, Fig. 6). Remaining five studies indicated significant effects of STN-DBS for reducing the dose compared with GPi-DBS (p < 0.00001, WMD = 287.59, 95% CI: 206.69 to 368.49, Fig. 6).

## Discussion

### Main findings

In the present multiple-treatments meta-analyses, there were evidences to support the hypothesis of treatment difference between GPi-DBS versus medical therapy (p < 0.05) and STN-DBS versus medical therapy (p < 0.05) in the treatment of parkinsonian symptoms in PD patients in terms of UPDRS scores. Meanwhile, we found that GPi-DBS had the similar efficacy on the UPDRS scores when compared with STN-DBS, suggesting that GPi-DBS and STN-DBS improved the clinical symptoms of PD equally well. In addition, this study also confirmed DBS (including GPi-DBS and STN-DBS) could significantly reduce PDQ-39 score in these subjects compared with medical therapy. However, improvement in PDQ-39 was greater after GPi-DBS than STN-DBS. In addition to improve the motor function and quality of life, the effect size of DBS was evident for the reduction of the required L-dopa medication dose compared with medical therapy. Consistent with previous results, STN-DBS allowed L-dopa medication dosages to be reduced to lower levels than GPi-DBS. Overall, the results from this paper showed that either GPi-DBS or STN-DBS was an effective technique to control patients’ symptoms and improve their functionality and quality of life. Nevertheless, the UPDRS scores measuring parkinsonian symptoms revealed no significant difference between GPi-DBS and STN-DBS.

These results were essentially identical to those described in previous review articles. Perestelo-Pérez *et al.*^14^ reported DBS (mainly STN-DBS, six RCTs) significantly improved patients’ symptoms, functionality and quality of life compared with best medical treatments. The effects sizes were intense for the reduction of motor signs and improvement of functionality in the off-medication phase. Moderate effects were observed in the case of motor signs and time in good functionality in the on-medication phase. In this meta-analysis, DBS (eight RCTs) of both targets (GPi and STN-DBS) was overall better than medical therapy in terms of UPDRS scores, PDQ-39 and LED with more participants. Liu Y *et al.*^30^ indicated that GPi and STN-DBS improved motor function for PD patients (six RCTs). Differences in therapeutic efficacy for PD were not observed between the two procedures. STN-DBS allowed greater reduction in medication for patients, whereas GPi-DBS provided greater relief from psychiatric symptoms. Similarly, we also showed GPi-DBS had the equal efficacy on the UPDRS scores when compared with STN-DBS with eight RCTs. PDQ-39 improvements were greater after GPi-DBS than STN-DBS, and STN-DBS allowed L-dopa medication dosages to be reduced to lower levels than GPi-DBS. In addition, we carried out a comprehensive overview of all RCTs that compared GPi-DBS, STN-DBS and medical therapy in one paper to provide a clinically useful summary of the results of the multiple-treatments meta-analyses that can be used to guide treatment decisions.

### Interpretation of the results

DBS is a well-established modality for the treatment of advanced PD. Recent studies have found DBS plus best medical therapy to be superior to best medical therapy alone for patients with PD and early motor complications, but its mechanism of action has been elusive^31^. To date, different mechanisms have been proposed to explain the therapeutic actions of DBS. Several studies in both experimental and clinical found that DBS could increase the output from the STN, as evidenced by the increased activity in the GPi, the downstream nucleus of the STN^32^. Therefore, a decoupling of the activities between the soma and the axon at the stimulation target may occur. In addition, recently, an important role of the motor cortex is becoming clear, and the emerging pictures have important implications for both the pathogenic process and treatment strategies for PD^5^. Moreover, site of surgery is a consideration when a surgical option is being considered. Our studies found no differences between GPi-DBS and STN-DBS in terms of improvement of UPDRS scores in line with previous meta-analyses^30^. STN-DBS allowed greater reduction in LED for patients, whereas GPi-DBS provided greater relief from quality of life (PDQ-39). To our knowledge, several studies of GPi-DBS versus STN-DBS also showed equivalent motor outcomes in a short-term follow up^33^. Concern has been expressed regarding the long-term durability of neurostimulation for PD. Meanwhile, the differences maybe due to the populations that were included, or differences related to the pharmacologic treatment of patients are treated. What is more, DBS is supposed to affect the firing rates and bursting patterns of neurons and, ultimately, the synchronized oscillatory activity of neuronal networks^34^. Both DBS targets can form such networks, which could explain why STN and GPi equally improved motor function. Increased understanding of the mechanism of DBS and the pathophysiology of the basal ganglia are needed to explain this issue.

Adverse effects of DBS consist of a wide variety of neurological and neuropsychological complications such as those related to surgery, hardware and stimulation. A recent extensive review suggests that the major surgery-related risk is intracranial hemorrhage and the overall incidence of hemorrhage was 5.0%, with symptomatic hemorrhage occurring in 2.1% of patients and hemorrhage resulting in permanent neurological deficit or death in 1.1%^35^. The most common hardware complications include infections, electrode migrations or misplacements, skin erosion, wire fractures and device malfunction, and the rate ranging from 4.3 to 17.8% between different studies. Stimulation-related adverse effects include muscle contractions, dysarthria, tremor, dyskinesia, headache and pain^36^. In general, DBS is a relatively safe approach associated with an encouragingly low rate of adverse effects. In addition, since DBS is expensive, cost must be considered when determining risk versus benefit^37^. A potential advantage reported in patients receiving DBS and medical therapy was significant reduction in medication use^20^. This would reduce both potential adverse effects and cost of the drugs used. What is more, long-term benefit of DBS must also be considered. Several studies have indicated that DBS does not alter the progression of PD. All in all, DBS is safe and soundness for advanced PD patients.

### Implication from this research

A recent report had detailed patient characteristics that were likely to predict a positive outcome to DBS. These characteristics included responsiveness to L-dopa, younger age, and no cognitive dysfunction or psychiatric diseases *et al.*^38^. Namely, preoperative L-dopa responsiveness was found to be predictive of average improvement in UPDRS scores following DBS surgery. As such, though it is definite that the relationship between preoperative L-dopa responsiveness and surgical outcome exists, the actual magnitude may be obscured^39^. In addition, longer disease duration was correlated with a higher UPDRS scores at baseline^40^. Consequently, there were also higher absolute UPDRS scores following surgery that were expected. Patients receiving DBS and best medical therapy were significant reduction in medication use had been validated by a multicenter study that evaluated the economic cost in PD subjects who underwent STN-DBS^41^. As efficacy of DBS is widely established, debate regarding when in the course of disease DBS surgery should be performed has arisen. The common approach is to provide surgery to patients when L-dopa has failed and all other options have been exhausted. Further studies would be required to evaluate when the optimum time window for DBS surgery to achieve maximum efficacy. Moreover, site of surgery is a consideration when a surgical option is being considered. Based on this paper, DBS can be employed to stimulate either STN or GPi in terms of improve UPDRS scores. The differences of PDQ39 and LED, if validated by other studies, could help determine the best site for DBS stimulation based on individual patient characteristics. One possibility reasons for this difference between the effects of DBS in GPi and STN over time is that the difference in doses of antiparkinsonian medications associated with STN and GPi stimulation is responsible^7^. The higher relative levels of dopaminergic medication taken by patients after GPi-DBS may enhance quality of life by improving aspects of PDQ-39 score. Thus, future study designed for research need to select suitable target for surgery according to the individual subject characteristics. Finally, stimulation-related side effects result mostly from diffusion of current into neighboring anatomic structures. In general, these side effects can be avoided by optimizing electrode placement. Hence, this needs to be carefully balanced in the future studies to ascertain the narrow window between the threshold for therapeutic effect and the appearance of side effects.

### Limitations

This paper has some potential weaknesses. Significant heterogeneity was observed in this analysis. A potential explanation may be that the measurements in each study were different. There were 3 studies included in the current study that contained larger numbers of patients (n > 200) than the other studies, which may create certain bias. Finally, we only included studies published in English, which may also create potential bias.

## Conclusion

Based on current available information, either GPi-DBS or STN-DBS plus medical therapy was superior to best medical therapy alone in terms of reducing UPDRS scores and improving quality of life and decreased medication requirements. Meanwhile, our results showed no differences between GPi-DBS and STN-DBS in either of our primary outcomes (UPDRS scores). STN-DBS was more effective for reduction in medication than GPi-DBS. Alternatively, GPi-DBS was more effective for improving the PDQ-39 score than STN-DBS.

## Methods

We undertook a systematic review and multiple-treatments meta-analysis based on the Preferred Reporting Items for Systematic Reviews and Meta-Analyses (PRISMA) Statement^42^. Meanwhile, to this study, we have no predefined protocol about the process and objective of this meta-analysis publication or available to the research community as an online appendix.

### Study selection and data collection

For our analysis, we only included RCTs that compared GPi-DBS versus STN-DBS, or GPi versus medical therapy, or STN-DBS versus medical therapy in the advanced PD patients. To identify the relevant studies, we electronically searched databases of PubMed, Google scholar and Cochrane Central Register of Controlled Trials (CENTRAL) electronic databases up to June 2015 for all English language publications. The search terms were “Parkinson” and “stimulation” to include more comprehensive literatures. Reference lists from the resulting publications and reviews were used to identify further relevant publications. The following inclusion criteria were used: 1) randomized, controlled, properly concealed, clinical trials, comparing STN-DBS with GPi-DBS or with medical therapy to treat idiopathic PD; 2) studies describing advanced PD patients with certain degree response motor fluctuations, dyskinesia, painful dystonia, or bradykinesia despite optimal pharmacological treatment; 3) studies that used the UPDRS to measure the baseline disease and post-treatment results; 4) reports in which outcomes were measurable continuous variables; 5) reports that were published in English. Studies and patient populations were excluded for the following reasons: 1) not RCTs studies; 2) no adequate data about UPDRS as the outcome measure; 3) DBS was performed in pathologies other than PD; 4) duplicate publications; or 5) data could not be extracted. For each study, information was carefully extracted from all the eligible studies, including (a) the first author, year of publication, number of subjects, sex ratio (male/female), mean age of subjects, duration, diagnostic criteria of PD and country of centers; (b) outcome measures such as UPDRS scores, PDQ-39 and LED; (c) intervention characteristics of the trial groups and control groups. Two persons within the reviewing team independently reviewed references and abstracts retrieved by the search, assessed the completeness of data abstraction, and confirmed the quality rating. We used a structured data abstraction form to ensure consistency of appraisal for each study (Table 1).

### Risk of bias of RCTs

The quality assessment of each RCT was assessed independently using the Cochrane Handbook for Systematic Reviews of Interventions^43^. Two investigators independently evaluated the methodological quality of the included studies. The tool classes studies as having low, moderate, or high risk of bias across six domains; sequence generation, allocation concealment, blinding, missing data, selective reporting and other biases. Disagreements were resolved through consensus or discussed with a third author.

### Statistical analysis

UPDRS as the primary outcome measure was considered as continuous data, and then an estimate of the combined effect sizes utilizing WMD was given, and its standard error with 95% confidence interval. Given the potential clinical or methodologic heterogeneity between multi-studies, a random-effects model was used, which yields a more conservative estimate of the pooled effect. If outcomes were presented from the studies at different time points, we extracted data from the last time point. If means and standard deviations were not provided, we calculated them from standard errors, CI, or other statistical indices. Results from intention-to-treat analysis were preferred over results from completer analyses. For the assessment of heterogeneity, the I^2^ statistic and chi-square tests were used. Standard meta-analyses were performed with Revman version 5.1. Probability value p < 0.05 were considered significant. The trials had to be conducted with populations of similar age and disease profile sharing a common treatment or placebo arm^44^. For example, a multiple-treatments meta-analysis to estimate the baseline risk or benefit associated with the administration of GPi-DBS versus medical therapy, STN-DBS versus medical therapy and GPi-DBS versus STN-DBS.

## Additional Information

**How to cite this article**: Xie, C.-L. *et al.* Effects of neurostimulation for advanced Parkinson’s disease patients on motor symptoms: A multiple-treatments meta-analysis of randomised controlled trials. *Sci. Rep.* **6**, 25285; doi: 10.1038/srep25285 (2016).

## Figures and Tables

**Table 1:** Basic characteristics of included studies.

Study	Criteria/Country	Basic data: M/F (n); age; duration		Intervention		Outcome measure
		Trial	Control	Trial	Control	
Jose 2001 [15]	Idiopathic PD/America	27/11(38), 55.7 ± 9.8y 15.6 ± 2.7y	60/36(96), 59.0 ± 9.6y 14.5 ± 1.3y	Bilateral GPi-DBS for 6 months	Bilateral STN-DBS for 6 months	1. UPDRS part II, III (on/off), 2. AE
Valerie 2005 [16]	Advanced PD (5 >HY >3)/Switzerland	NR (10), 54 ± 12y 10.3 ± 2y	NR (10), 61 ± 9y 15.6 ± 5y	Bilateral GPi-DBS for 12 months	Bilateral STN-DBS for 12 months	1. UPDRS part II, III (on)
Günther 2006 [17]	Advanced PD /Germany and Austria	50/28(78), 60.5 ± 7.4y 13.0 ± 5.8y	50/28(78), 60.5 ± 7.8y 13.8 ± 5.6y	Bilateral STN-DBS for 6 months	Medical therapy for 6 months	1. UPDRS part II, III (on/off), 2. PDQ-39 3. LED
Schupbach 2007 [18]	Idiopathic PD (3 ≥HY ≥2)/America	5/5(10), 48.4 ± 3.3y 7.2 ± 1.2y	5/5(10), 48.5 ± 3.0y 6.4 ± 1.1y	Bilateral STN-DBS for 18 months	Medical therapy for 18 months	1. UPDRS part II (on/off), III, IV (on) 2. PDQ-39
Witt 2008 [19]	Idiopathic PD/Germany	36/24(60), 60.2 ± 7.9y 13.8 ± 6.3y	41/22(63), 59.4 ± 7.5y 14.0 ± 6.1y	Bilateral STN-DBS for 6 months	Medical therapy for 6 months	1. UPDRS part I, II (on/off), III (on/off), IV 2. PDQ-39, 3. LED
Frances 2009[20]	Idiopathic PD (HY >2)/America	98/33(121), 62.4 ± 8.8y 10.8 ± 5.4y	110/24(134), 62.3 ± 9.0y 12.6 ± 5.6y	Bilateral GPi–DBS for 6 months	Medical therapy for 6 months	1. UPDRS part I, II, III, IV (on) 2. PDQ-39, 3. LED
Laura 2009[21]	Idiopathic PD/America	16/6(22), 61.3 ± 5.5y 12.3 ± 3.6y	14/6(20), 61.3 ± 9.0y 14.3 ± 3.9y	Unilateral GPi-DBS for 6 months	Unilateral STN-DBS for 6 months	1. UPDRS III (on/off), 2. LED 3. PDQ-39
Adrian 2010 [22]	Advanced PD (4 >HY >2)/UK	125/58(183), 59(37–79)y 11.5(2.0–32.2)y	135/48(183), 59(36–75)y 11.2(1.0–30.0)y	Bilateral GPi-DBS for 1 y	Medical therapy for 1 y	1. UPDRS part I (on), II, III, total (on/off) 2. PDQ-39, 3. AE
Kenneth 2010 [23]	Advanced PD (HY >2)/America	133/19(152), 61.8 ± 8.7y 11.5 ± 5.4y	116/31(147), 61.9 ± 8.7y 11.1 ± 5.0y	Bilateral GPi-DBS for 24 months	Bilateral STN-DBS for 24 months	1. UPDRS part III (on/off), I, II, IV (on) 2. PDQ-39, 3. LED
Angelo 2011 [24]	Advanced PD (HY >3)/Italy	8/13(21), 51 ± 8y NR	7/12(19), 49 ± 11y NR	STN-DBS for 5 y	Medical therapy for 5 y	1. UPDRS III (on), 2. LED
Lee 2011 [25]	Idiopathic PD/America	12/1(13), 65.5 ± 8.6y 15.1 ± 10.2y	13/1(14), 63.8 ± 6.3y 16.8 ± 6.2y	Bilateral GPi-DBS for 6 months	Bilateral STN-DBS for 6 months	1. UPDRS III (on/off)
Laura 2012[26]	Idiopathic PD/Italy	13/1(14), 61.1 ± 8.4y 12.9 ± 10.17y	11/4(15), 61.4 ± 5.5y 11.9 ± 4.8y	Bilateral GPi-DBS for 6 months	Bilateral STN-DBS for 6 months	1. UPDRS III (on), 2. LED
Frances 2012 [27]	Advanced PD (HY >2)/America	77/12(89), 60.4 ± 8.3y 11.4 ± 4.9y	56/14(70), 60.7 ± 8.9y 11.3 ± 4.7y	Bilateral GPi-DBS for 36 months	Bilateral STN-DBS for 36 months	1. UPDRS part I, II (on), III, IV (on/off) 2. PDQ-39, 3. LED
Michael 2012 [28]	Advanced PD/America	63/38(101), 60.6 ± 8.3y 12.1 ± 4.9y	21/14(35), 59.5 ± 8.2y 11.7 ± 4.1y	Bilateral STN-DBS for 12 months	Medical therapy for 12 months	1. UPDRS part III (on/off), I, II, IV (on) 2. LED
Schuepbach 2013 [29]	Advanced PD/Germany and France	94/30(124), 52.9 ± 6.6y 7.3 ± 3.1y	85/42(127), 52.2 ± 6.1y 7.7 ± 2.7y	Bilateral GPi-DBS for 2 y	Medical therapy For 2 y	1. UPDRS part II, III, IV (on), 2. PDQ-39 3. LED
Vincent 2013 [10]	Advanced PD (5 >HY >2)/Netherlands	44/21(65), 59.1 ± 7.8y 10.8 ± 4.2 y	44/19(63), 60.9 ± 7.6y 12.0 ± 5.3y	Bilateral GPi-DBS for 12 months	Bilateral STN-DBS for 12 months	1. CMDAE, 2. UPDRS part II, III (on/off), 3. LED

Abbreviations: HY: Hoehn and Yahr stage; CMDAE: cognitive, mood, and behavioural adverse effects; UPDRS: Unified Parkinson’s Disease Rating Scale; LED: Levodopa equivalent dose; PDQ-39: Parkinson’s disease Questionnaire; NR: no report; Y: years.

**Table 2:** The methodological quality of included studies.

Study	A	B	C	D	E	F	Total
Jose 2001 [15]	+	+	+	+	+	?	5
Valerie 2005 [16]	+	+	+	+	+	?	5
Günther 2006 [17]	+	+	−	+	+	+	5
Schupbach 2007 [18]	+	+	−	+	+	?	4
Witt 2008[19]	+	+	+	+	+	+	6
Frances 2009 [20]	+	+	+	+	+	+	6
Laura 2009[21]	+	+	−	+	?	?	3
Adrian 2010 [22]	+	+	+	+	+	+	6
Kenneth 2010 [23]	+	+	+	+	+	+	6
Angelo 2011 [24]	+	−	+	+	?	?	3
Lee 2011 [25]	+	+	+	+	+	?	5
Laura 2012[26]	+	+	+	+	?	?	4
Frances 2012 [27]	+	−	+	+	+	?	4
Michael 2012 [28]	+	+	−	+	+	+	5
Schuepbach 2013 [29]	+	+	+	+	+	+	6
Vincent 2013 [10]	+	+	+	+	+	+	6

A: Sequence generation; B: Allocation concealment; C: Blinding of participants, personnel and outcome assessors;

D: Incomplete outcome data; E: No selective outcome reporting; F: Other sources of bias; +: Yes; −: No; ?: Unclear.
